# Sports activities and mental distress in young people in deprived urban areas in South America: a cross-sectional analysis

**DOI:** 10.1186/s13104-025-07288-y

**Published:** 2025-07-01

**Authors:** Sofia Madero, Luis Ignacio Brusco, Francisco Diez-Canseco, Carlos Gómez-Restrepo, Natividad Olivar, Pablo Ezequiel Flores-Kanter, Sumiko Flores, Ana L. Vilela-Estrada, Ángela Flórez-Varela, M. Esteban Uribe Pinto, Diliniya Stanislaus Sureshkumar, Catherine Fung, Stefan Priebe

**Affiliations:** 1 Department of Psychiatry and Mental Health, School of Medicine, Universidad de Buenos Aires, Buenos Aires, Argentina; 2 CRONICAS Center of Excellence in Chronic Diseases, Universidad Peruana Cayetano Heredia, Lima, Perú; 3 Department of Psychiatry and Mental Health, Pontificia Universidad Javeriana, Bogotá, Colombia; 4 Department of Clinical Epidemiology and Biostatistics, Pontificia Universidad Javeriana, Bogotá, Colombia; 5 San Ignacio University Hospital, Bogotá, Colombia; 6 Unit for Social and Community Psychiatry, Wolfson Institute of Population Health, Queen Mary University of London, London, UK; 7 Centre for Psychosocial Medicine, University of Hamburg, Hamburg, Germany

**Keywords:** Adolescents, Young adults, Anxiety, Depression, Sports activities, Mental health

## Abstract

**Objective:**

To explore the association between sport activities and depression/anxiety symptoms among young people in disadvantaged urban areas of South America.

**Results:**

Out of a total of 2375 participants, 1131 (47.1%) had engaged in sports in the previous month. Sports activities, particularly those performed with others, were associated with lower anxiety and depression. There was a trend for this association to be stronger in male than in female participants.

**Supplementary Information:**

The online version contains supplementary material available at 10.1186/s13104-025-07288-y.

## Introduction

In South America, depression and anxiety are among the most prevalent mental disorders among adolescents and young adults [1]. In major cities across the region young people are exposed to a range of risk factors for mental health disorders, including poverty, violence, availability of illegal drugs and significant social inequalities. These challenges are particularly pronounced for those residing in deprived neighbourhoods [2]. Research indicates a strong correlation between living in socially disadvantaged areas and negative mental health outcomes [3].

An international literature suggests that engaging in sports and physical activities can significantly improve the mental health of young people. A study conducted in deprived areas reported an association of participation in physical and sports activities with lower symptoms of depression and increased happiness [4]. Sports activities not only provide young people with opportunities for peer interaction but can also serve to alleviate stress and build self-confidence [5]. At least 60 min of daily physical activity have been linked with lower levels of depression and anxiety in youth [6]. Key factors for this appear to be both physical activity and social relationships [7].

A study in the United States found that team sport, as opposed to not participating in any sport, was linked to several positive outcomes, including a 10% reduction in anxiety and depression, a 17% drop in social problems, and a 20% reduction in rule-breaking behaviour [8]. The benefits of team sports may stem from the social support and connectedness fostered through shared activities [9].

A further study indicated that involvement in sports clubs was associated with lower rates of depressive symptoms, and suggested that social interactions at the sports club may be just as significant as the sports activity itself. People who participated in sports activities such as sports clubs, gyms, or independent exercise had a lower risk of experiencing depression than those who did not engage in regular exercise [10]. A 2 year longitudinal study also found that higher levels of sports participation were associated with fewer depressive symptoms over time, suggesting a protective effect of regular sports engagement during adolescence [11].

Despite the existing evidence for a positive impact of sports on mental health, research focusing specifically on deprived urban youth in South America remains scarce [12]. This study aims to assess whether participation in sports activities is linked with symptoms of depression and anxiety in young people living in deprived urban neighbourhoods in South America. We assessed levels of symptoms, and not formal diagnoses, to reflect a dimensional approach of mental distress and to be independent of diagnostic systems that tend to change over time.

## Methodology

### Study design

This cross-sectional study was conducted as part of the broader research programme with the title “Building resilience and resources to reduce depression and anxiety in young people from urban neighbourhoods in Latin America (OLA).” This programme aimed to identify resources and activities that may help young people to prevent and recover from depression and anxiety in low-resource urban settings in South America [12, 13].

### Setting

This cross-sectional investigation assessed participants in three major South American capitals: Buenos Aires (Argentina), Bogotá (Colombia), and Lima (Peru). A consistent strategy was created in these cities to identify deprived areas and recruit participants. The recruitment took place between April 2021 and November 2022. For selecting areas, the United Nations Development Programme’s Human Development Index was used in Bogotá and Lima, and the Unsatisfied Basic Needs Index in Buenos Aires [14]. In each city, we selected areas within the lower 50% of these rankings. In these communities, participants were identified through schools, local arts centres, youth organisations, and various community and educational organisations.

### Participants

The study included two age groups within the range of youth as defined by the United Nations as ranging from 15 to 24 years of age: adolescents (15–16 years old) and young adults (20–24 years old) [15]. Participants were included if they resided in the defined geographic areas and were capable of providing informed consent or assent for young people under 18 years old. Parental or guardian informed consent was required for those under 18 years of age. Individuals with severe mental illnesses, cognitive impairments, or illiteracy were excluded from the study.

### Variables of interest and measurements

Each participant completed either a paper or online battery assessing symptoms of depression and anxiety as well as sports activities in the last month (see supplementary file 1) and socio-demographic data (see supplementary file 2). The protocol for the OLA research programme with the overall approach and the recruitment and assessment methods has been described elsewhere [13].

### The patient health questionnaire-8 (PHQ-8) and the general anxiety disorder-7 (GAD-7)

The PHQ-8 [16–19] and GAD-7 [20–23] are widely established and validated self-reported scales for assessing symptoms of depression and anxiety. On eight or seven items respectively, they ask about symptoms in the last 2 weeks. Responses are given on 4-point Likert scales (0 = ‘‘not at all’’ to 3 = ‘‘every day’’).

### Sports activities

Sports activities were assessed with a series of self-report questions. First, participants were asked if they had regularly participated in any sport or physical activity, either in person or online in the last 30 days, excluding compulsory school sports, with ‘‘Yes’’ or ‘‘No’’. Secondly, they selected the sports they regularly participated in from a list including Football, Cycling, Basketball, Going to the gym, Volleyball, Tennis, Swimming, Martial arts, Running, Skating, Athletics, and ‘‘Other (specify)’’. They were asked to identify up to three sports activities they had practiced. For each sports activity they reported, they were then asked whether they performed these activities ‘‘Alone,’’ ‘‘With friends,’’ or ‘‘Other (specify)’’ (multiple answers possible), and about the frequency of the activity, with five options ranging from ‘‘Less than once a week’’ to ‘‘Four or more times a week.’’

### Data analysis

For the analysis, we considered the first sports activity the participant reported as well as the information obtained by the follow up questions about who they conducted the sports activities with (alone or with others) and the frequency. For the category “sports with others” we combined the options “friends” and “others”.

Descriptive analyses were conducted for all included variables. For assessing the association of sports activities with symptoms of depression and anxiety, we computed linear regression models with symptom levels as dependent variables. We also assessed how the frequency of specific sports activities was associated with symptom levels.

Since the Shapiro–Wilk normality test based on the distribution of the residuals from the linear model was significant (W = 0.98, p-value < 0.001), we also applied robust linear models using an M estimator, by iterated re-weighted least squares [24]. All regression models were adjusted for gender and age group of participants.

In the regression models we tested interaction effects with gender and age group.

Since the linear regression models do not provide the actual size of difference in symptom levels between participants with and without sports activities, we added t-tests for independent samples with Welch's correction for unequal variances between groups. Moreover, we conducted t-tests to compare symptom levels in participants who did sports alone or not, and with others or not. Finally, if an interaction effect was found in the regression analyses, t-tests for independent samples were used to show the directions of the effect. All data analyses were calculated using the R programming language [25] and the Rstudio interface [26].

### Patient and public involvement

At the start of the study, we set up a Lived Experience Advisory Panel (LEAP) in the three cities, made up of young people aged 15 to 24 who had experienced mental health challenges. The LEAP met two to three times per year to provide feedback on the study's progress and future plans, which helped improve recruitment and data collection methods.

Arts organisations were engaged as partners to involve young people in activities aimed at gathering their insights on mental health. The insights gained from these activities, as well as focus groups with adolescents, young adults, and professionals, were used to shape the study’s data collection tools. All the gathered information was used for create arts-based methods for sharing the study’s findings.

## Results

### Participants

Out of a total of 2402 participants recruited, 2375 responded to all questions on symptoms and sports activities required for this analysis and constituted the sample for analyses presented in this study. The total sample included 1085 (45%) adolescents and 1317 (55%) young adults. A total of 1560 (65%) participants from the total sample were female, 815 (34%) were male, 24 (1%) answered the “other gender” option, and 3 were missing cases because they did not respond to the gender question.

### Symptoms and sports activities

The mean scores of the total sample were 8.52 (SD = 4.93) for anxiety, and 10.00 (SD = 5.97) for depression. Among the total sample, 1131 (47.1%) participants had practiced sports in the previous month. From the 1115 participants who answered the question with whom they performed the sports activities, 346 (31.0%) responded only alone, 726 (65.1%) only with others (friends/other), and 43 (4%) both alone and with others.

With respect to the type of sport, football was the most practiced sport (n = 492, 44%), followed by running (n = 367, 32%), going to the gym (n = 276, 24%), cycling (n = 174, 15%), basketball (n = 150, 13%), volleyball (n = 142, 13%), physical exercise—including home workouts—(n = 114, 41%), dancing (n = 60, 22%), athletics (n = 51, 4.5%), skating (n = 54, 4.8%), swimming (n = 40, 3.5%), and martial arts (n = 46, 4.1%); 135 (12%) participated in sports activities less than once per week, 237 (21%) once a week, 242 (22%) twice a week, 228 (20%) three times a week, and 280 (25%) four or more times a week.

### Symptoms in participants with and without sports activities

The findings of the regression analyses are shown in Tables 1 and 2.

**Table 1:** Regression analysis with sports activities as the independent variable and symptoms of depression as the dependent variable, adjusted for gender and age group

	β	Std. Error	t	p
(Intercept)	10.85	0.76	14.18	<0 .001
Sport Activity (Yes)	− 0.86	0.24	− 3.48	<0 .001
Gender (Female)	2.26	0.25	8.72	<0 .001
Gender (Other)	6.12	1.21	5.05	<0 .001
Age	− 0.10	0.03	− 2.83	< .0.01

Multiple R-squared: 0.053, Adjusted R-squared: 0.051

**Table 2:** Regression analysis with sports activities as the independent variable and symptoms of anxiety as the dependent variable, adjusted for gender and age group

	*β*	Std. Error	*t*	*p*
(Intercept)	7.41	0.63	11.74	< 0.001
Sport Activity (Yes)	-0.61	0.20	− 2.99	< 0.01
Gender (Female)	1.97	0.21	9.24	<0 .001
Gender (Other)	4.89	0.99	4.89	< 0.001
Age	0.002	0.03	0.08	0.93

Multiple R-squared: 0.051, Adjusted R-squared: 0.050

The linear models with the frequency of sports activities showed a statistically significant association with symptoms of depression but not with anxiety (see supplemental file 3). The results of the robust linear models did not alter any of the findings (see supplemental file 4).

The symptom levels of participants with and without any type of sports activities, with and without sports activities alone, and with and without sports activities with others are shown in Table 3.

**Table 3:** Symptoms of anxiety and depression scores among participants practicing and not practicing sport activities in the previous month

Parameter	Sports activity	Mean (± SD)		*t*	*p*
		No	Yes		
Anxiety	Any Sport	9.04 (± 4.90)	7.93 (± 4.88)	5.58	< 0.001***
	Sports alone	8.57 (± 4.93)	8.27 (± 4.91)	1.13	0.258
	Sports with others	8.89 (± 4.90)	7.76 (± 4.90)	5.26	< 0.001***
Depression	Any Sports	10.66 (± 6.02)	9.29 (± 5.84)	5.62	< 0.001***
	Sports alone	10.13 (± 6.03)	9.45 (± 5.65)	2.13	0.033*
	Sports with others	10.40 (± 5.96)	9.22 (± 5.94)	4.50	< 0.001***

Values with * denote statistical significance at the p<0.05

* → p <.05 (moderately significant)

*** → p <.001 (very highly significant)

The scores of participants who practiced any sports were a bit more than one scale point lower for both anxiety and depression. Similar differences were found between participants with and without sports activities with others, whilst there was no significant difference between participants with and without sports activities alone.

When testing the interaction effects between sports activities in the last month and gender as well as age group on symptoms of anxiety and depression, only the interaction effect between sports activities and gender on depression showed a trend toward statistical significance (β = -0.906, p = 0.066). We therefore conducted post-hoc tests on the differences in depression levels between participants practicing and not practicing sports activities, separately for the male and female participants. The results are shown in Table 4.

**Table 4:** *-* Sports activity and symptoms of depression levels in male and female participants

Parameter	Sports activity	Mean (± SD)		*t*	*p*
		No	Yes		
Depression in male participants	Any sports	9.24 (± 5.95)	7.82 (± 5.50)	3.36	<0 .001***
Depression in female participants	Any sports	10.99 (± 5.95)	10.52 (± 5.84)	1.51	0.131

Values with * denote statistical significance at the p<0.05

*** → p <.001 (very highly significant)

The difference in symptoms of depression between male participants doing and not doing sport activities was almost 1.5 scale points and statistically significant, whilst the same difference was much smaller and not statistically significant among female participants.

## Discussion

### Main findings

The findings of this large study in deprived areas in South America show a clear link between sports activities and lower symptom levels of depression and anxiety among young people. The results showed that those adolescents and young adults who had performed sports activities in the last 30 days had fewer symptoms of anxiety and depression than those who had not performed sports activities.

In general, our findings align with previous literature that has shown the importance of sports as a protective factor for mental well-being in adolescents and young adults [27]. In addition, our results extend to the youth population in South America.

One may assume that the positive effects of sports may extend beyond physiological benefits and emphasise the benefits of sports activities with others, probably allowing for social support and connectedness.

Whilst sport activities with others appears more helpful than sport activities alone, there may be a gender difference with a strong impact in male young people but less so in females.

### Comparison against the literature

The findings are consistent with similar observations in the literature suggesting an association between sports activities and better mental health [28, 29]. Our finding that sports with others may be even more beneficial aligns with some reports in the literature. Previous research has suggested that anxiety and depression are more common in those young people who practice individual sports than in those who participate in team sports [30]. Football as a typical team sport was the most commonly practiced sport in our study.

The literature suggests that sports have been shown to be beneficial for individuals dealing with mental disorders [31]. Yet, many of the studies testing this have serious methodological limitations, and the studies vary significantly in terms of the population studied, the types of activities, and the instruments for assessing outcomes [32].

Our finding of a gender difference with benefits of sports activities for male but not female participants is inconsistent with previous reports of more positive effects in women as compared to men [8]. One can only speculate as to whether the more positive effect in men in our study is linked to the specific setting of deprived urban areas or the culture in South America or other reasons.

## Strengths and limitations

This is an extensive study conducted in the very challenging context of deprived areas in three large South American cities [12]. The study used established methods for assessing symptoms and avoided diagnostic categories that may change over time. The data collection was based on contacts with trained researchers. Moreover, the study assessed not only participation in sports in general but also the type of sport activity, and whether it was conducted alone or with others.

However, this study also has some limitations. Firstly, during some phases of the data collection, social restrictions because of the COVID-19 pandemic were in place. The restrictions varied over time and between countries and could have affected both sport activities and symptoms.

Secondly, the information provided by participants may have been subject to memory bias and social desirability bias. Thirdly, the sample was not representative which may have influenced the findings on levels of sport activities and symptoms more than their association, since associations are usually more robust against a selection bias.

Finally, this is a cross-sectional study, and the association do not establish evidence for a causal relationship.

## Conclusions

This was a cross-sectional study, and the association is likely to be bi-directional: young people with less anxiety and depression tend to perform more sports, and sports may help to reduce anxiety and depression. Also, the differences in symptoms between participants with and without sports activities are not of a magnitude to suggest that sports activities alone could be the answer to widespread anxiety and depression among young people. However, one may conclude that participation in sports is likely to have some benefits for the mental health of young people, benefits that may be important for a substantial number of individuals and are likely to be relevant on a public health level. Young people participating in sports activities more frequently had lower symptoms of depression, but there was no significant link of the frequency of activities with levels of anxiety symptoms. In the interpretation of this inconsistent finding, one should consider that most of the young people with sports activities were relatively frequently engaged in sports. Two thirds of them conducted sports activities at least twice per week, and an increase of frequency among such regular sports participants may not have much influence anymore.

Future research may seek to disentangle the possibly complex association between sport activities and mental distress, considering skills, practical options, and reward mechanisms. The findings of this study suggest that there may be relevant gender differences in the setting of deprived urban areas. It needs to be explored what the reasons for these differences are and whether they apply to other contexts, too.

Public health initiatives should consider strategies to promote consistent sports participation among adolescents and young adults. There is a need to invest in facilities and organisations in poor urban neighbourhoods so that young people have the opportunity to practice sports activities with others. Sports programmes should be tailored to these population needs, considering their poor access to resources such as football fields and other expensive tools. Building football fields in deprived areas may motivate adolescents and young adults to play (without travelling) and organise free football tournaments. This plan could be achieved by building networks between the school’s youth communities, sports clubs, or social clubs. Finally, policies should encourage young people to practice group sports, as part of healthy lifestyles, as regularly as possible.

Given that sports activities, particularly team sports, are often low-cost, they can present a realistic option for improving young people’s mental health in deprived areas where other resources and interventions are scarce. Nevertheless, despite the low cost, sports activities require space and opportunities that public health may want to provide.

## Supplementary Information

Supplementary material 1. Sports activities questionnaire.

Supplementary material 2. Socio-demographic questionnaire.

Supplementary material 3. Linear models with the frequency of sports activities and symptoms of depression and anxiety as dependent variables.

Supplementary material 4. Robust linear models for tables 1 and 2.

## Data Availability

The dataset analysed during the current study will be available beginning 9 months and ending 36 months following article publication to researchers on reasonable request from Professor Victoria Bird. Contact details: v.j.bird@qmul.ac.uk.

## Abbreviations

PHQ-8: Patient health questionnaire-8
GAD-7: General anxiety disorder-7
DSM-IV: The diagnostic and statistical manual of mental disorders
SD: Standard deviation
