# Supplementary figures and images for: Sports activities and mental distress in young people in deprived urban areas in South America: a cross-sectional analysis
Source: BMC Res Notes. 2025 Jul 1;18:257. doi: 10.1186/s13104-025-07288-y (PMC12217355; doi:10.1186/s13104-025-07288-y)

Supplementary file 1
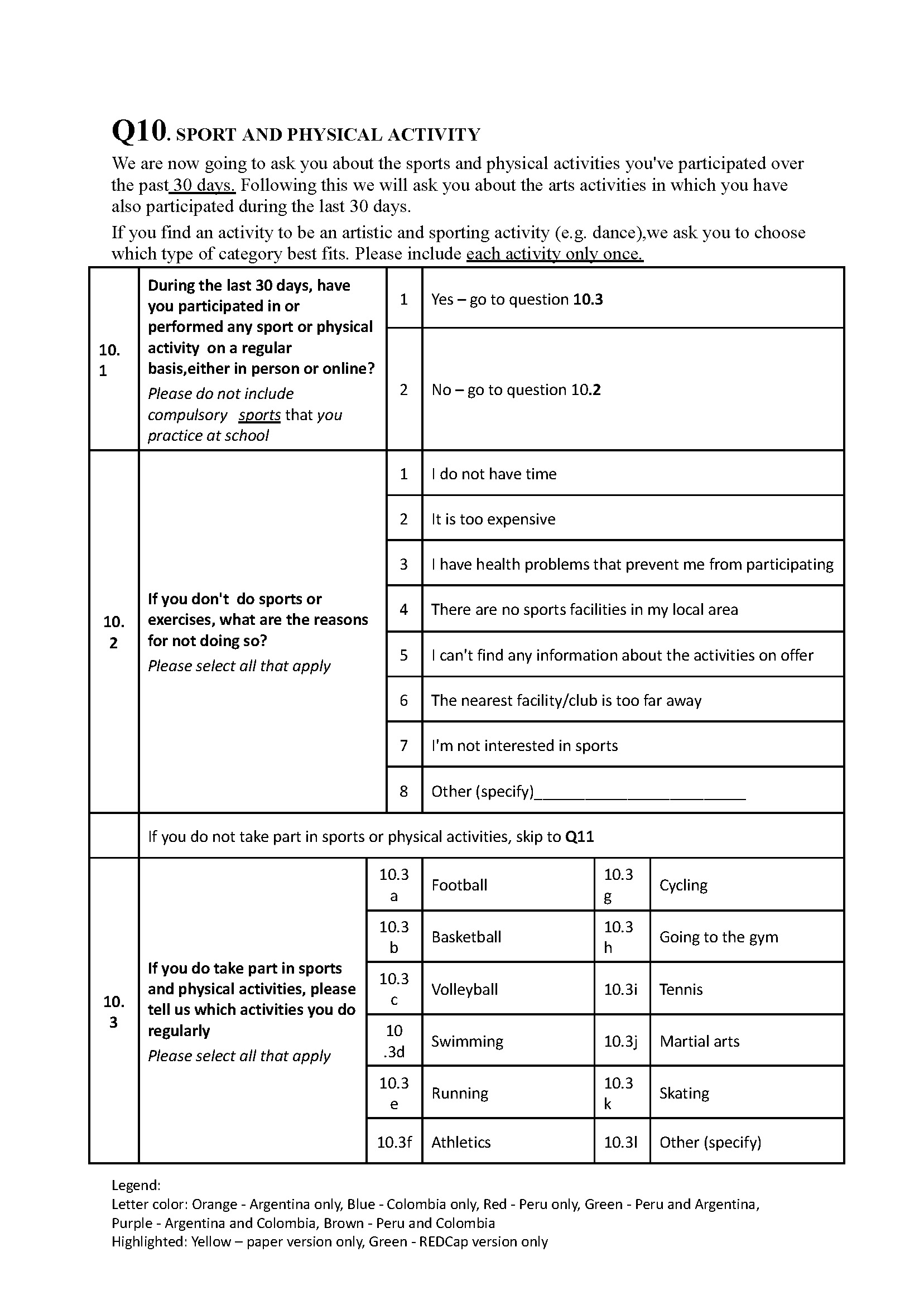


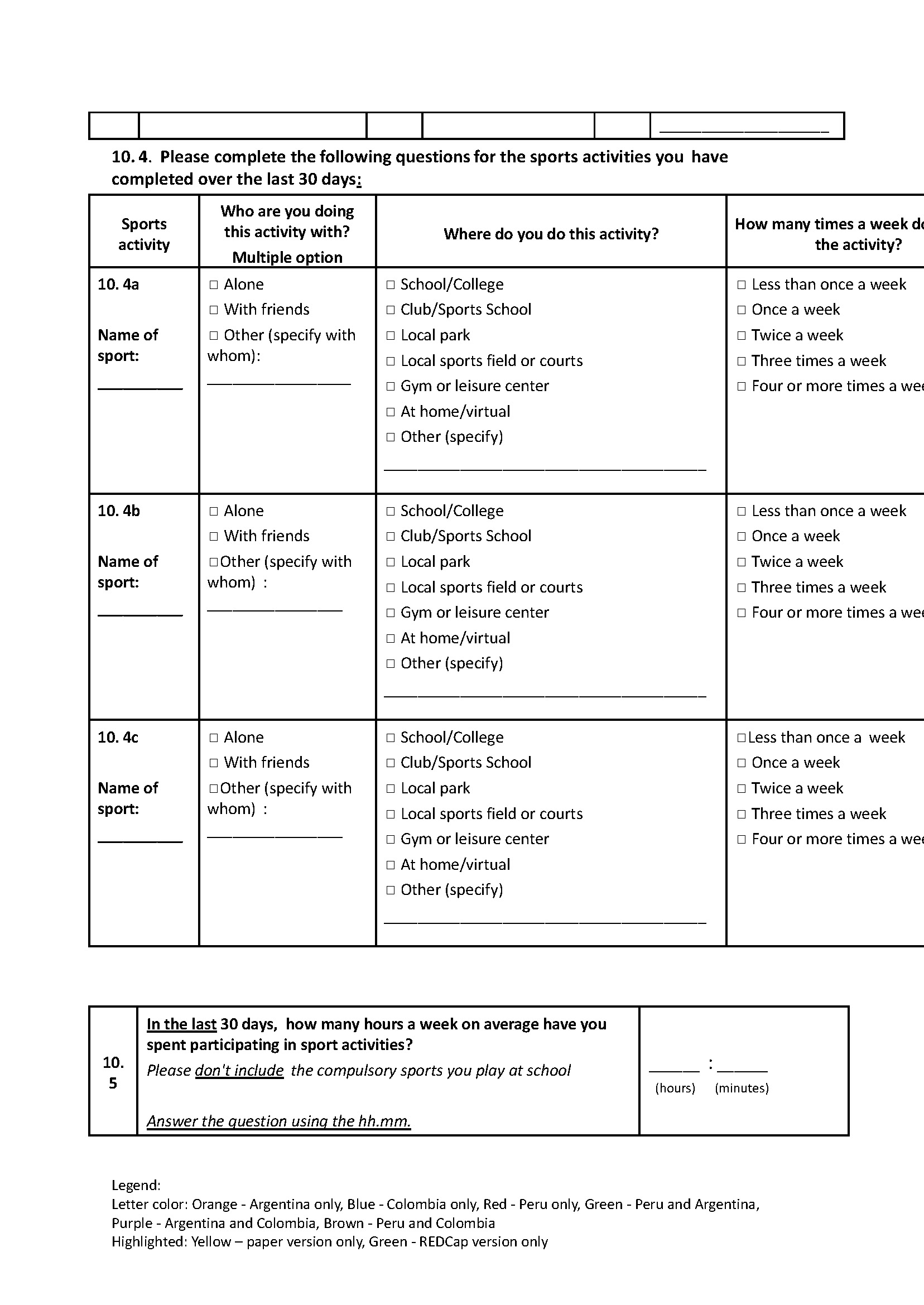


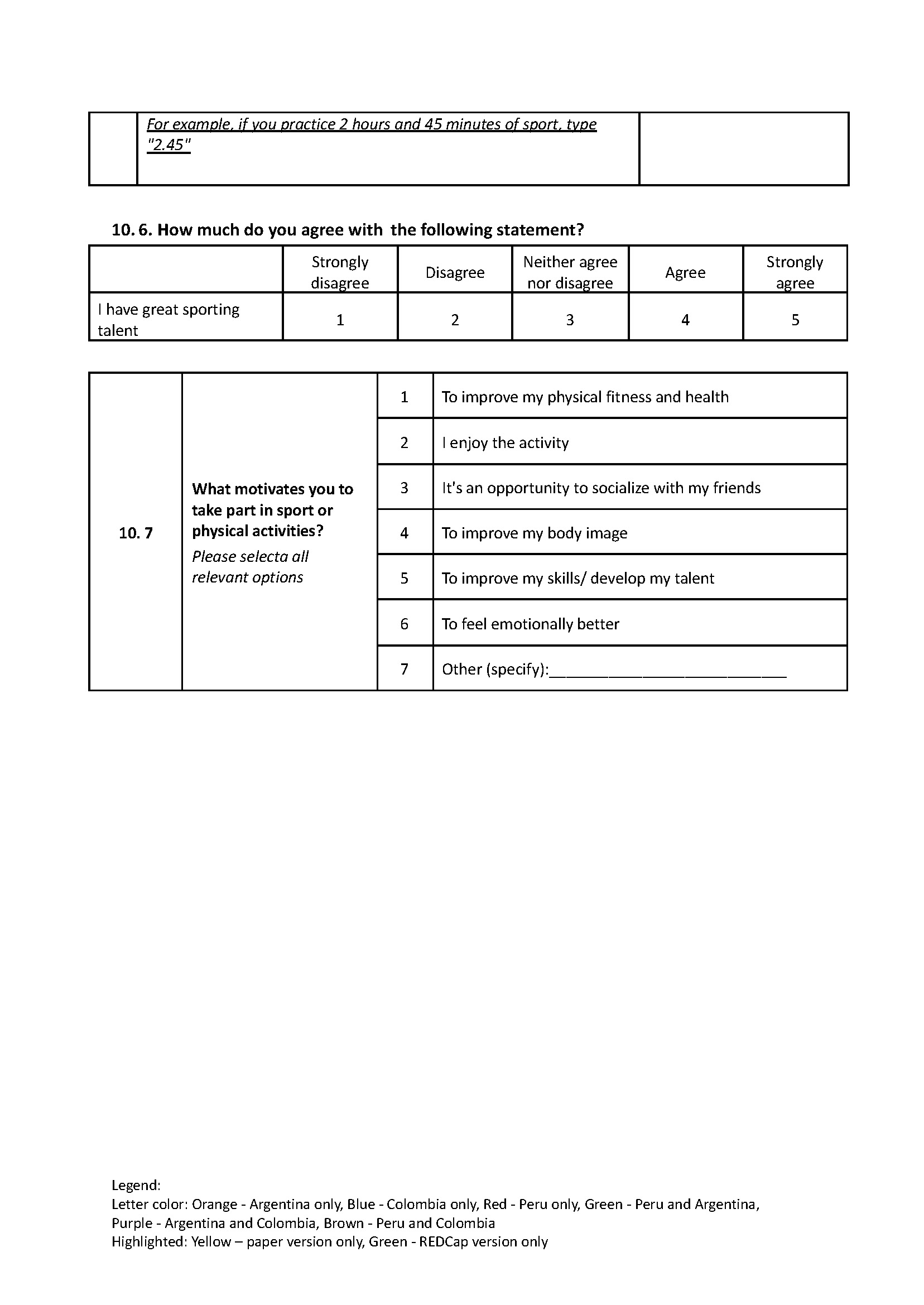

Supplement: Supplementary file 1 — Supplementary material 1. Sports activities questionnaire. [file 13104_2025_7288_MOESM1_ESM.docx]

Supplementary file 2


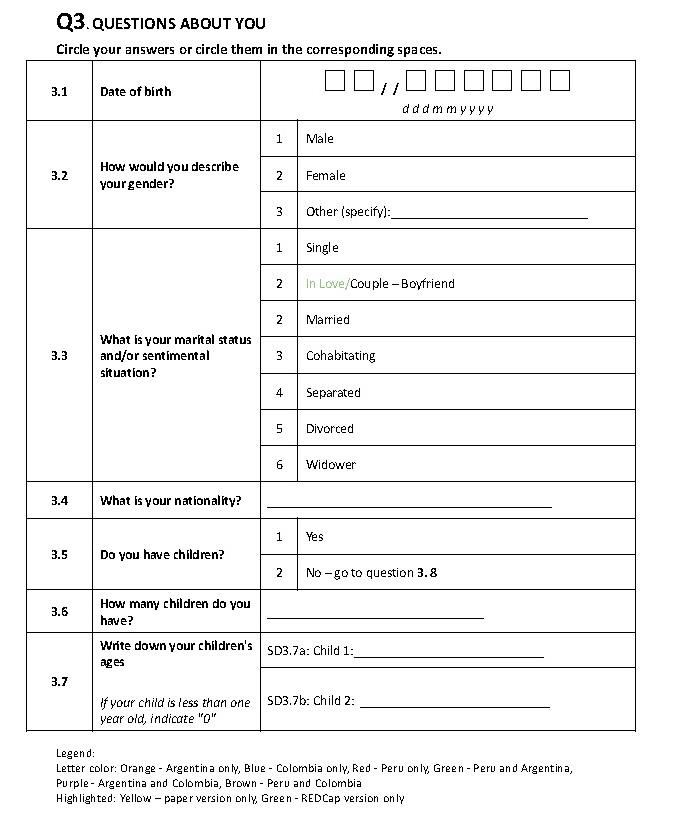


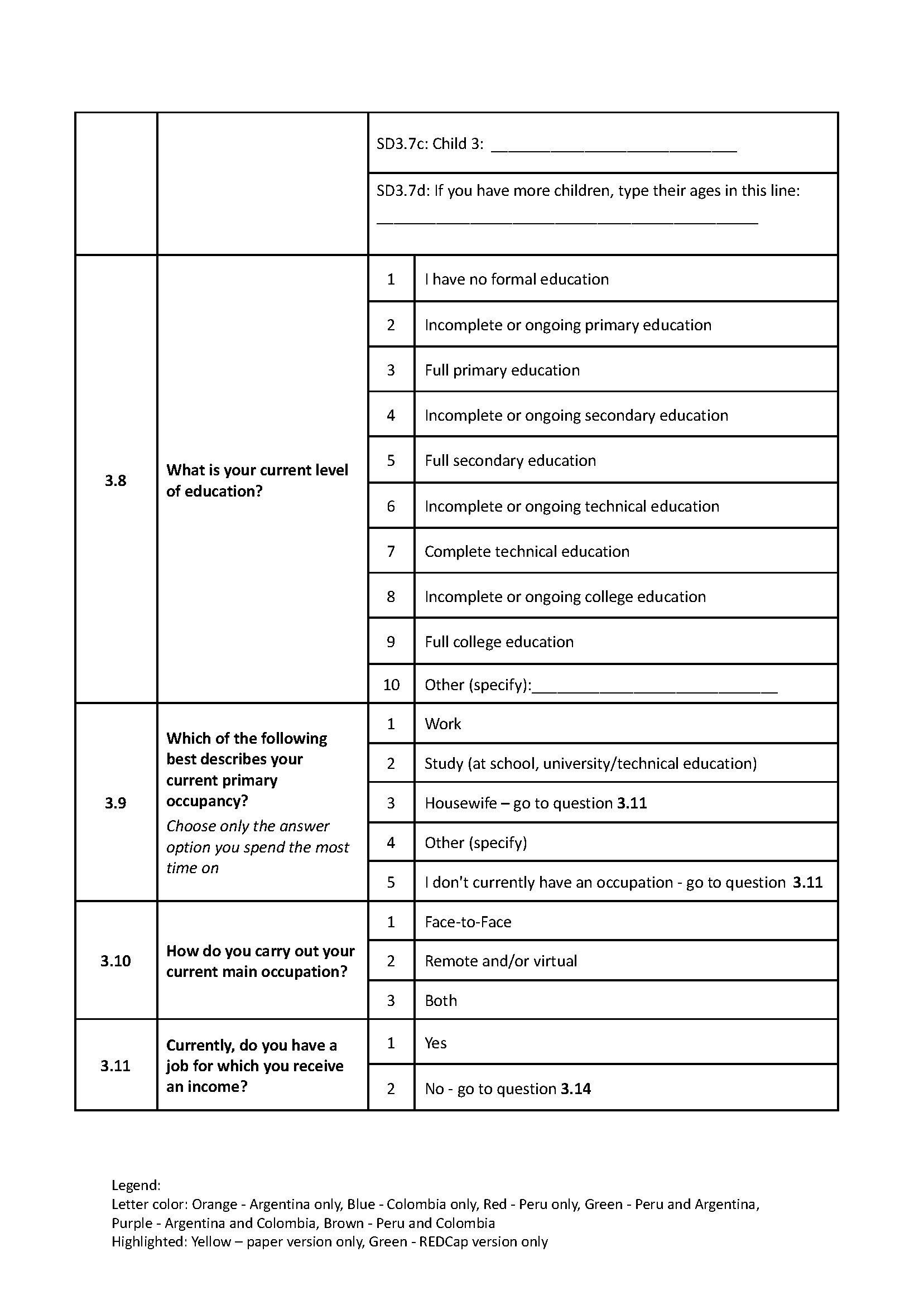


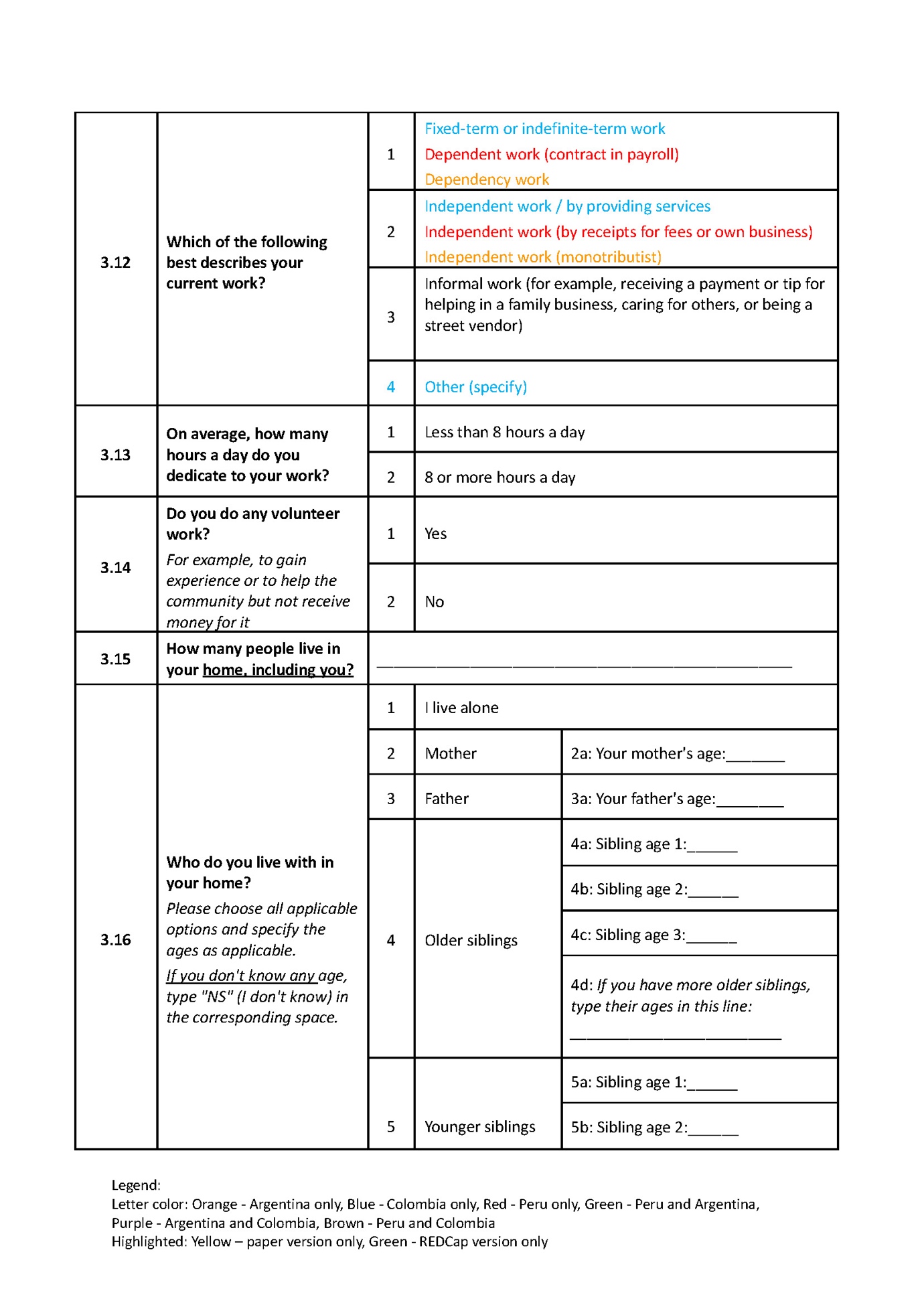


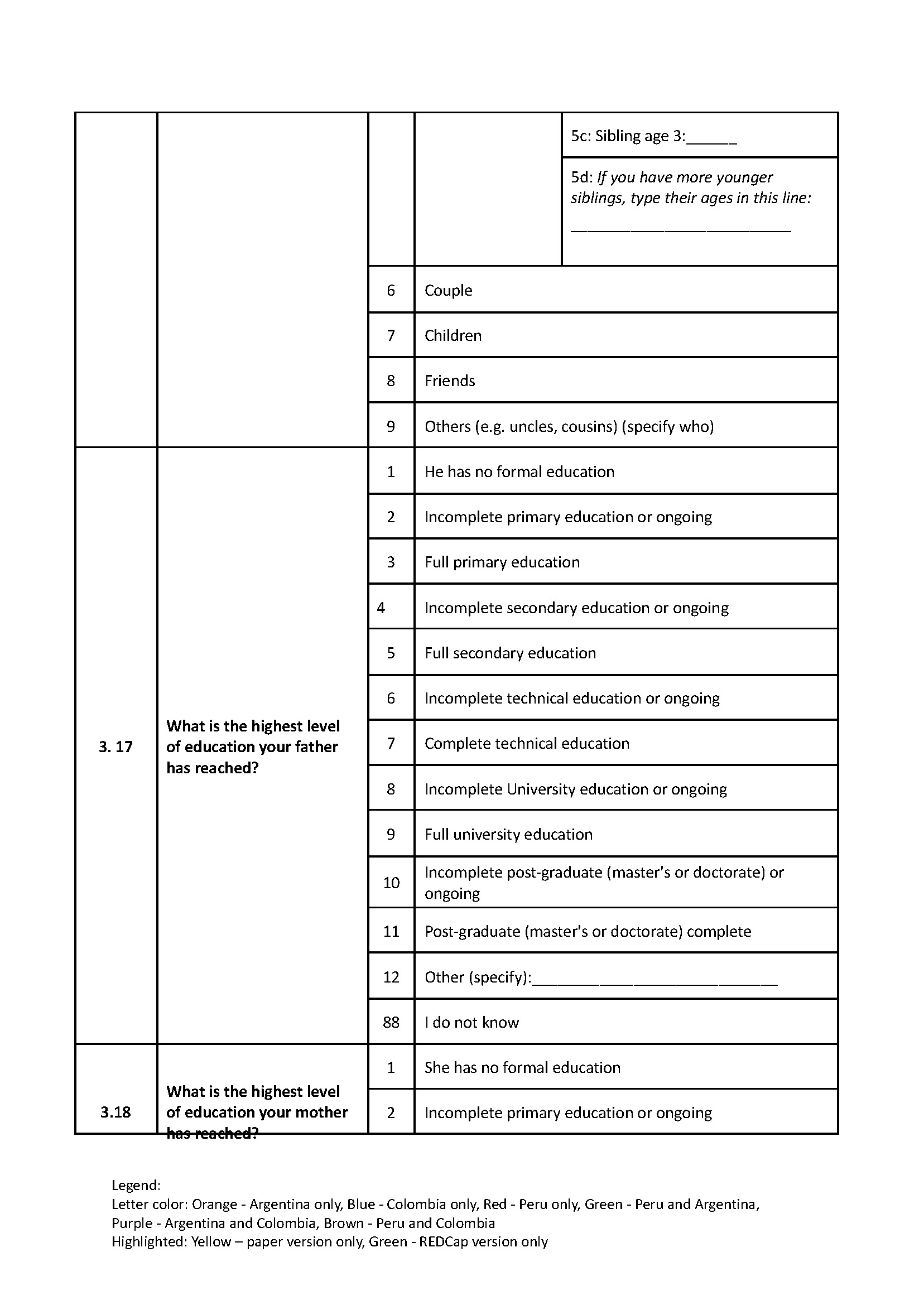


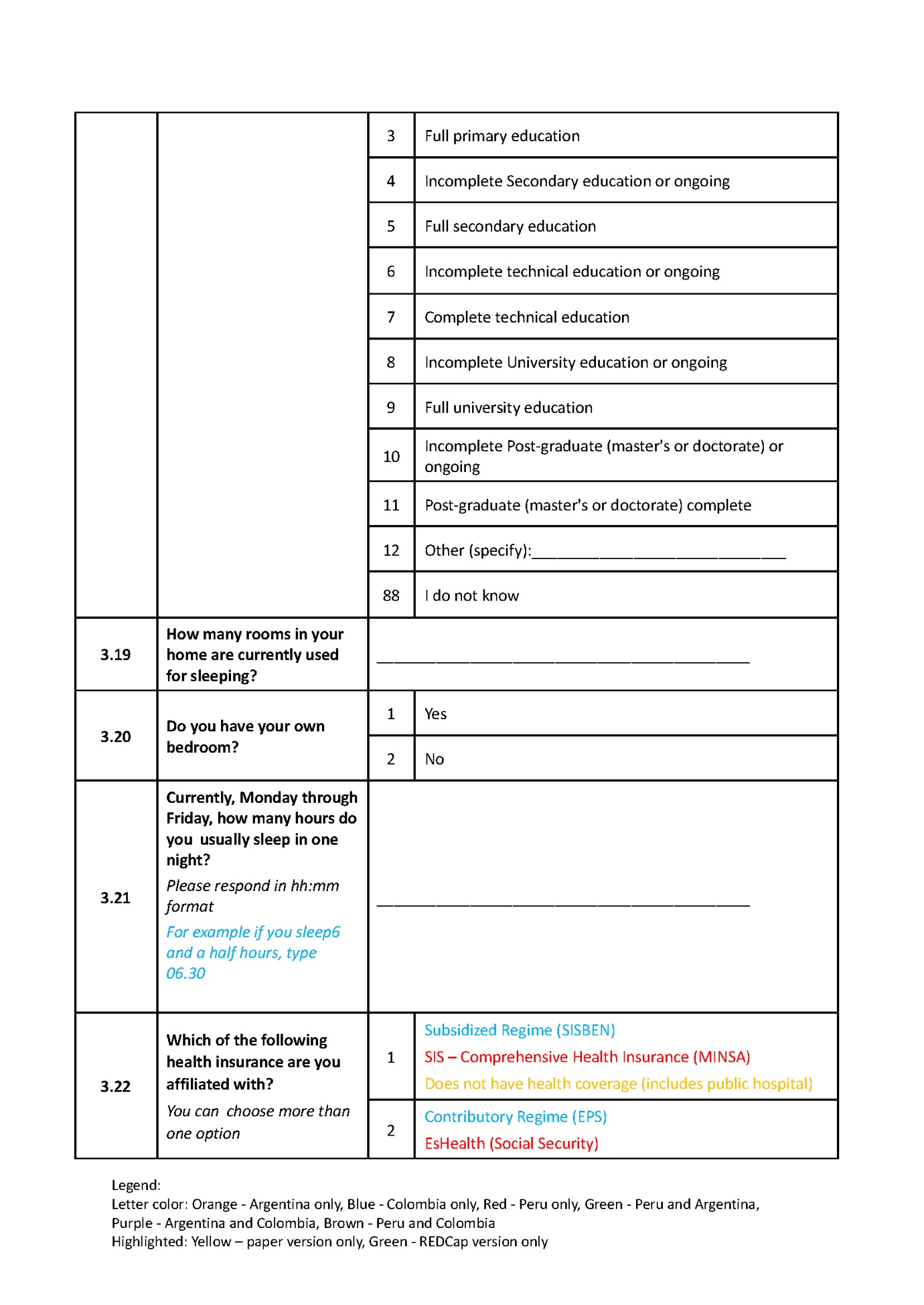


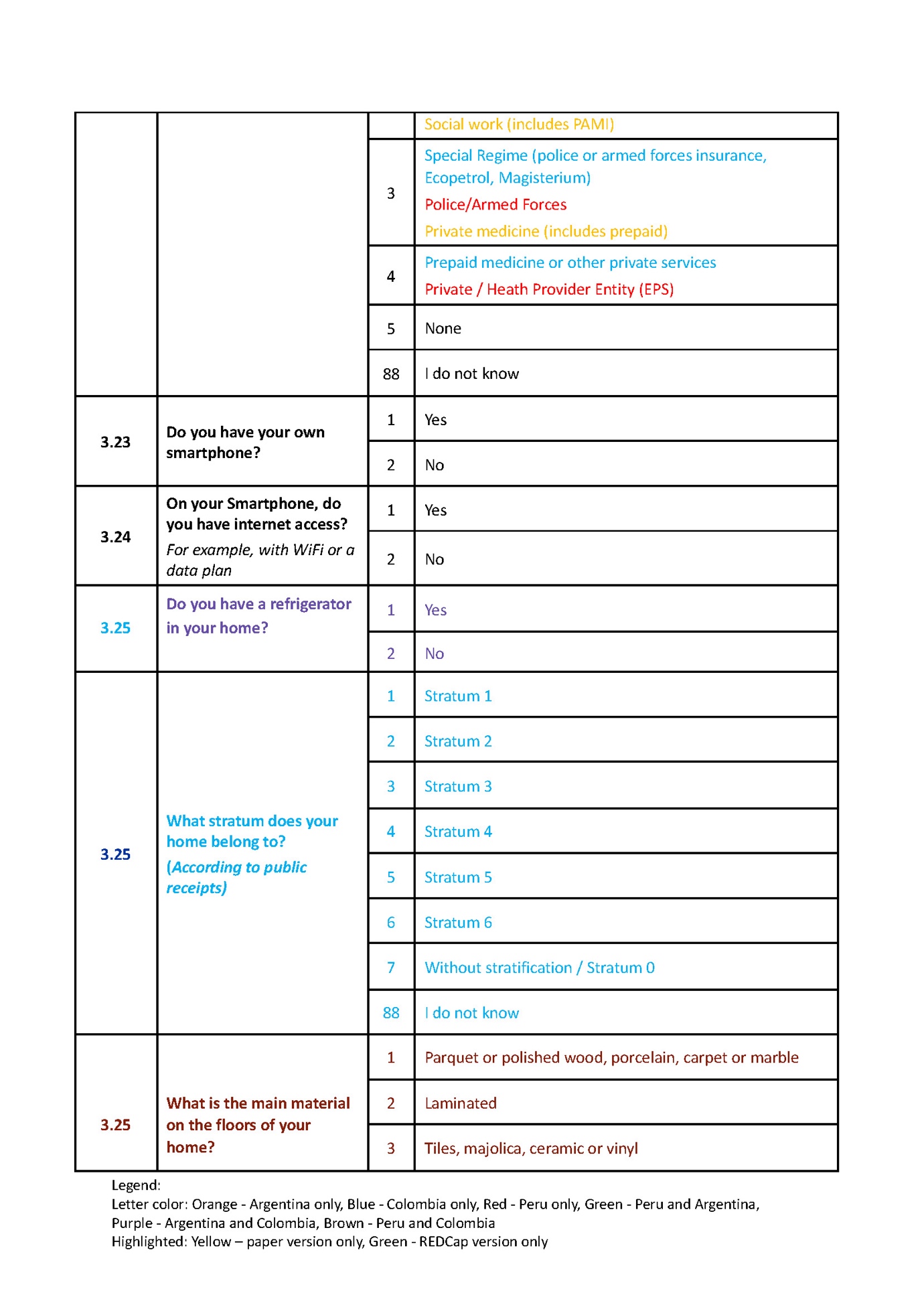


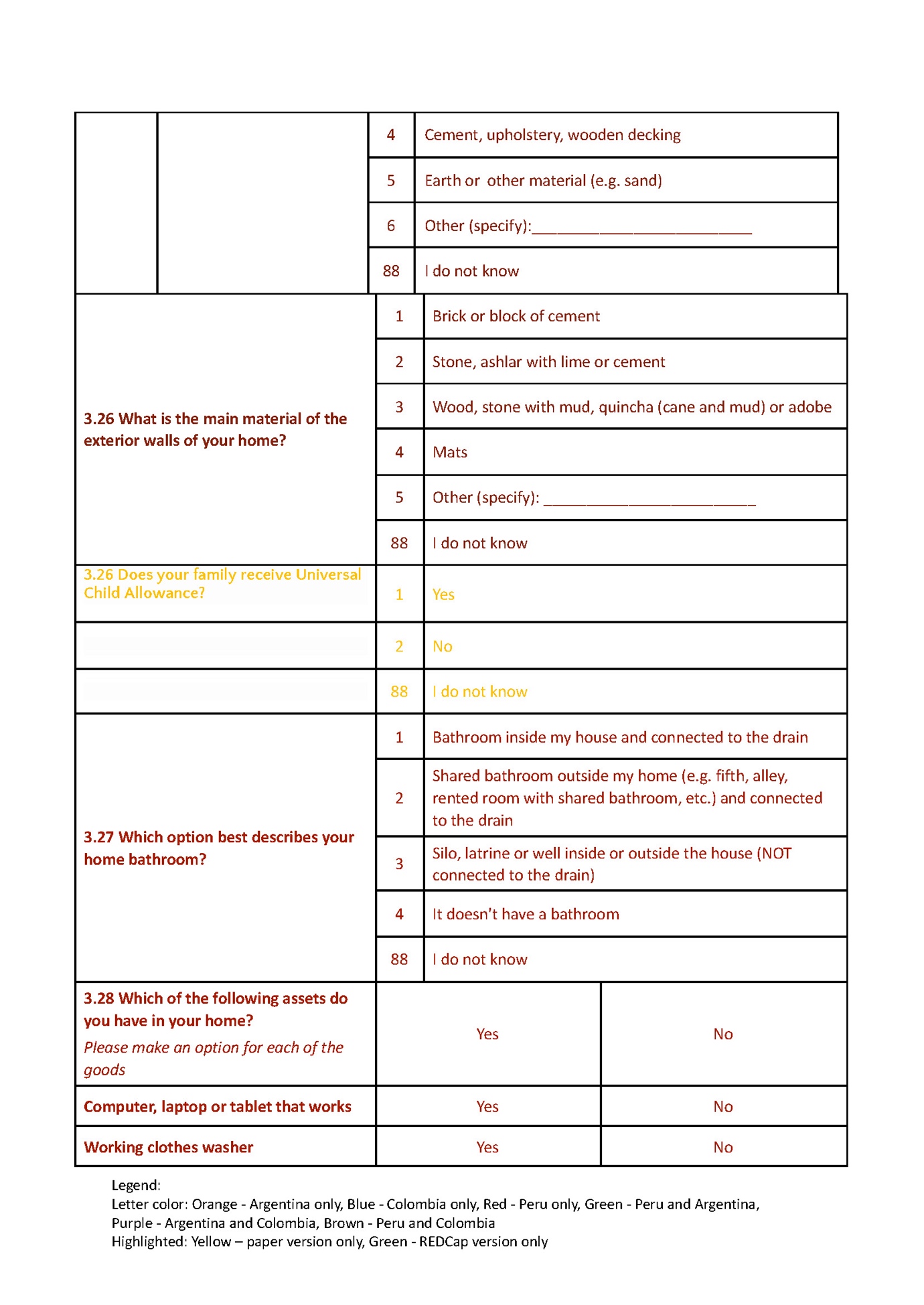


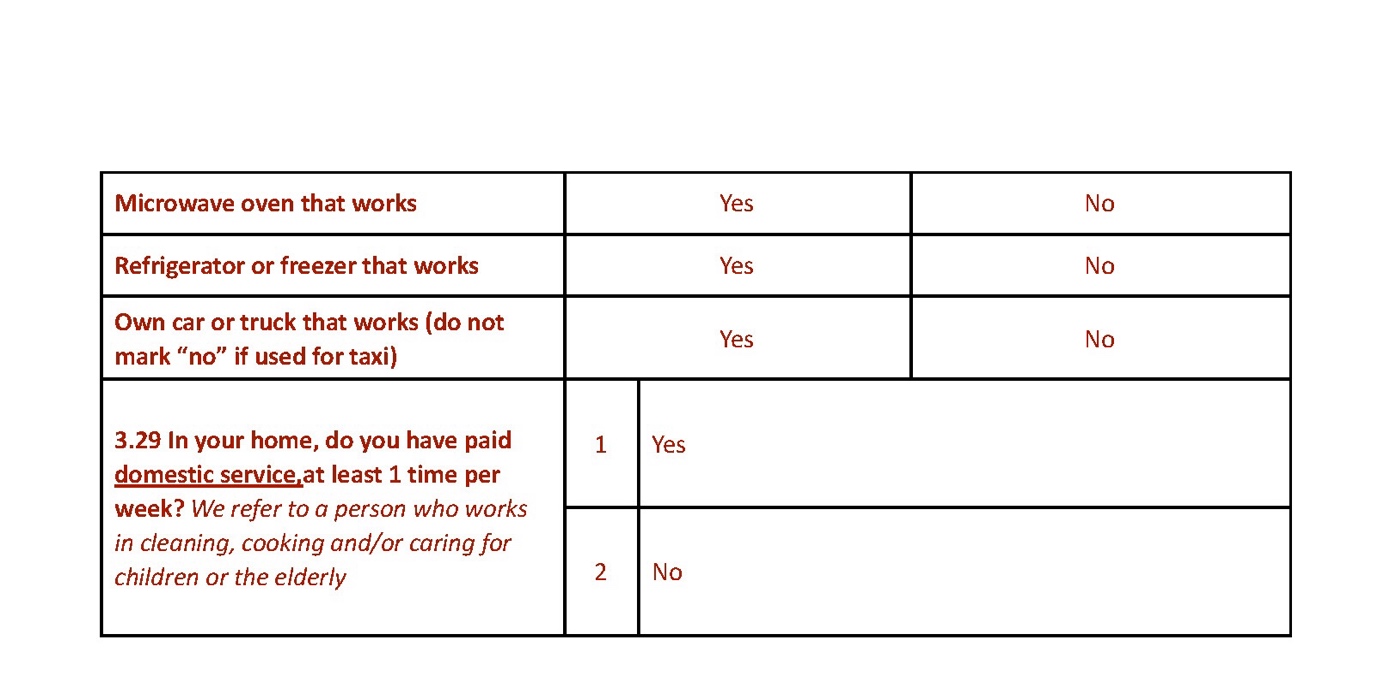

Supplement: Supplementary file 2 — Supplementary material 2. Socio-demographic questionnaire. [file 13104_2025_7288_MOESM2_ESM.docx]
