# Supplementary material for: Sports activities and mental distress in young people in deprived urban areas in South America: a cross-sectional analysis
Source: BMC Res Notes. 2025 Jul 1;18:257. doi: 10.1186/s13104-025-07288-y (PMC12217355; doi:10.1186/s13104-025-07288-y)
Supplement: Supplementary file 3 — Supplementary material 3. Linear models with the frequency of sports activities and symptoms of depression and anxiety as dependent variables. [file 13104_2025_7288_MOESM3_ESM.docx]

Supplementary file 3

Linear models with the frequency of sports activities and symptoms of depression and anxiety as dependent variables.

Depression - Linear model with the frequency of sports activities as independent and symptoms of depression as dependent variables.

|  | Estimate | Std. Error | t | Pr(>\|t\|) |  |
| --- | --- | --- | --- | --- | --- |
| (Intercept) | 9.34585 | 1.13158 | 8.259 | 4.14e-16 | *** |
| Frequency | -0.30727 | 0.12764 | -2.407 | 0.01623 | * |
| Gender (female) | 2.58142 | 0.34555 | 7.471 | 1.61e-13 | *** |
| Gender (other) | 7.55134 | 2.86076 | 2.640 | 0.00842 | ** |
| Age | -0.02493 | 0.05303 | -0.470 | 0.63837 |  |

Adjusted R-squared: 0.05924; Normality Test: ,98 p<,05

Robust:

|  | *β* | Std. Error | t |
| --- | --- | --- | --- |
| (Intercept) | 9.3156 | 1.1870 | 7.8478 |
| Frequency | -0.3304 | 0.1339 | -2.4677 |
| Gender (female) | 2.7014 | 0.3625 | 7.4524 |
| Gender (other) | 7.7458 | 3.0010 | 2.5811 |
| Age | -0.0323 | 0.0556 | -0.5812 |

Anxiety- Linear model with the frequency of sports activities as independent and symptoms of anxiety as dependent variables.

|  | Estimate | Std. Error | t | Pr(>\|t\|) |  |
| --- | --- | --- | --- | --- | --- |
| (Intercept) | 6.41533 | 0.94862 | 6.763 | 2.19e-11 | *** |
| Frequency | -0.17819 | 0.10717 | -1.663 | 0.09668 | . |
| Gender (female) | 2.18409 | 0.28992 | 7.533 | 1.02e-13 | *** |
| Gender (other) | 6.98731 | 2.39824 | 2.914 | 0.00365 | ** |
| Age | 0.04941 | 0.04447 | 1.111 | 0.26679 |  |

Adjusted R-squared: 0.0565; Normality Test: ,97 p<,05

Robust:

|  | *β* | Std. Error | t |
| --- | --- | --- | --- |
| (Intercept) | 6.1361 | 0.9632 | 6.3703 |
| Frequency | -0.1927 | 0.1088 | -1.7711 |
| Gender (female) | 2.2999 | 0.2944 | 7.8128 |
| Gender (other) | 7.2136 | 2.4352 | 2.9623 |
| Age | 0.0537 | 0.0452 | 1.1888 |
