# Supplementary material for: Sports activities and mental distress in young people in deprived urban areas in South America: a cross-sectional analysis
Source: BMC Res Notes. 2025 Jul 1;18:257. doi: 10.1186/s13104-025-07288-y (PMC12217355; doi:10.1186/s13104-025-07288-y)
Supplement: Supplementary file 4 — Supplementary material 4. Robust linear models for tables 1 and 2. [file 13104_2025_7288_MOESM4_ESM.docx]

Supplementary file 4

Robust linear models for tables 1 and 2.

Robust linear model for Table 1 - Regression analysis with sports activities as independent and symptoms of depression as dependent variable, adjusted for gender and age group.

|  | *β* | Std. Error | t |
| --- | --- | --- | --- |
| (Intercept) | 10.78 | 0.80 | 13.32 |
| Sport Activity (Yes) | -0.88 | 0.26 | -3.38 |
| Gender (Female) | 2.39 | 0.27 | 8.71 |
| Gender (Other) | 6.48 | 1.28 | 5.05 |
| Age | -0.11 | 0.03 | -2.85 |

Robust linear model for Table 2 - Regression analysis with sports activities as independent and symptoms of anxiety as dependent variable, adjusted for gender and age group.

|  | *β* | Std. Error | t |
| --- | --- | --- | --- |
| (Intercept) | 7.14 | 0.66 | 10.70 |
| Sport Activity (Yes) | -0.62 | 0.21 | -2.88 |
| Gender (Female) | 2.11 | 0.22 | 9.33 |
| Gender (Other) | 5.12 | 1.05 | 4.84 |
| Age | 0.004 | 0.032 | 0.12 |
